# Opium in Fevers and Inflammations

**Published:** 1854-07

**Authors:** H. R. Payne

**Affiliations:** Marshal, Ill.


THE NORTH-WESTERN

### MEDICAL AND SURGICAL JOURNAL.

NEW SERIES.

VOL. III. JULY, 1854. NO. 7.

### ORIGINAL COMMUNICATIONS.

ART. I.—*Opium in Fevers and Inflammations.*—Read before the Æsculapian Medical Society, by H. R. PAYNE, M.D., Marshal, Ill., May 31st, 1854.

IT is not my design on this occasion, to attempt to present all of the indications for the use of this valuable medicine, as we possess no agent the uses of which, on many points, are so generally settled in the minds of the Profession.

My object will be attained, if I present in a clear and satisfactory manner, some of the more recent views held by some of our most eminent men; with the addition of my own reflections, which I have formed from a limited experience.

In the Profession of Medicine, as well as in the attainment of other knowledge, we should not be too hasty in condemning recent views, for such always retards the progress of science; on the other hand, we can expect to make but little advance in elevating the standard of our profession, if we too readily acquiesce in the views of others. But, as our desire should be truth, and the good of our fellow-men, we should carefully and candidly observe and investigate for ourselves, before we come to any settled conclusion.

In the study of this subject I hope I have no other object in view, and as my own investigations are far from being thorough, I shall expect to be generously indulged by the members of this Society.

Opium, as I have before said, is a remedy, the uses of which in many respects, is settled in the minds of the Profession. Centuries ago its virtues were acknowledged, and it may be almost traced to the days of Hippocrates himself. It is now perhaps more extensively used than any one agent we possess, as there is scarcely any disease in which at one stage or another of its progress it is not indicated. Let us then examine some of the indications that give rise to its use: 1st, its primary operation is that of a stimulant; 2d, it is sedative; 3d, anti-spasmodic; 4th, diaphoretic; 5th, promotes rest; 6th, relieves pain; and 7th, as recent investigations go to show, controls inflammatory action.

For example, what stimulant is better adapted for those low forms of Nervous or Typhoid Fever, where the vital forces are weakened, and the patient sinking from an exhausting Diarrhœa, as small and repeated portions of Opium?

Or what remedy so surely assuages the pangs of parting life, in the last stage of Pulmonary Consumption, when the patient is so harrassed with the Cough, Expectoration, and Diarrhœa?

What remedy shows its anti-spasmodic propensities in Spasmodic Colic so fully, as large portions of Opium?

Or what remedy is more extensively used, and possesses greater virtues in that grave disease Puerperal Fever, than Opium?

Dr. Armstrong, a sound practical teacher and writer, was a great advocate for the use of Opium in this disease; he gave it in all cases in which the local pain, and constitutional disturbance were prominent. But it is unnecessary for me to multiply examples in which its powers are acknowledged; these are familiar to you all.

My object, as before stated, is to present it in that light, in which recent investigations have gone far to prove it should stand.

I am not however, one of those that would advocate its indiscriminate use, or so wild and imaginative as to sustain it when my own judgement has not tried and approved of the remedy—

but, that it deserves a higher place in the list of remedial agents, in certain forms of disease, I believe.

In the treatment of Typhus and Typhoid Fevers as a curative agent, its claims are strongly argued. Perhaps there is no one more strongly advocates its claims in these forms of Fever than Prof. Austin Flint, of the University of Louisville, and formerly of Buffalo, who has tested its efficacy in these, as well as in other diseases, and in whose sound judgement and extensive practical information, is to be placed great reliance. He uses it early and in large portions in what he calls the Abortive Treatment, and frequently with the effect of putting an end to the disease.

If it fails in this he does not suspend its use, but gives it when the symptoms of Coma Vigilance make their appearance.

He does not stop here, but uses it in those grave cases attended with violent Delirium, combined with Tart. Ant. et Potassæ, as he considers that in the most of these cases there is no inflammatory tendency.

Dr. Henry of Springfield, Illinois, speaks in strong terms of the use of opium in large portions, in continued Fevers; he, as Dr, Flint, does not use it to the exclusion of other remedies, but considers them as auxilaries, and Opium as the "Sheet anchor." He takes the same view of the subject as Dr. Flint, he says, "In our Typhoid forms of Fever, I believe the affection of the brain is most generally the result of diminished power, and is only to be relieved by sedative and stimulant remedies. It may be that it is on this principle, that a large dose of Opium and quinine so generally relieves the tendency to Coma and even Coma itself in this type of Fever." It must not however be supposed that, because both of these able men advocate the claims of opium in Coma vigilance, and even in true coma itself that they favor its use in true Cerebral affections, when actual Inflammation exist, as they consider it in such cases contra-indicated.

In speaking of the virtue of opium in continued fevers Dr. Henry observes, "I regard the use of opium when given in the way I propose, that is, combined with Calomel and in large doses, as the most safe and efficient remedy, in all forms of continued fever, to be found in the whole list of our remedial agents." He then gives the difference of Treatment in what he calls the Mercurial, when Mercury is made the basis, (the plan he formerly pursued;) And the opiate course, as the result of this test he says " In the cases treated on the Mercurial plan, I find that the average number of days attendance was about eight, and the mortality one in twenty-seven. Under the opium plan of treatment, the average number of days attendance has been about four, and the mortality not one in ninety." Here is a vast difference in the result, and how is it to be accounted for, if not on account of the superior efficacy of opium ? It certainly was not because the diseases were milder, as he states ; it includes "all the forms of Fever from the mildest Bilious remittents, to the most malignant forms of Conjestive and Typhus.

In cases of Inflammation, the claims of opium are strongly urged. Formerly the opinion was maintained, and still is, by many Physicians that its use is contra-indicated in such cases, particularly when of a high grade. This view of the subject at first sight is rational, as it is well known that opium given in small portions operates directly as a stimulant, and when given in such quantities, would necessarily increase the fever, as well as the Local Inflammation ; but this view does not destroy the proposition that opium is not an Antiphlogistic, on the contrary experience has shown, that when given in large portions it operates reversely to that given in smaller portions, producing copious diaphoresis, with a reduction in the action of the fever. Dr. Stokes in his valuable Practice of Medicine, gives his testimony in its favor; he says, "some time since I made a series of Clinical experiments, with the view of ascertaining the power which opium possessed in relieving Inflammation, and the result has been that in many cases where the powers of life are so low that we cannot have recourse to the lancet, or any kind of depletory measures, opium alone furnishes us with a powerful means of subduing inflammatory action." Again, it is a fact, and stated upon the authority of Dr. Henry, in one of his propositions, that a large dose of opium produces less constipation of the Bowels than a smaller dose.

Here then we see the necessity of modifying the remedy in different diseases, or in different stage of the same disease, as a large portion has a far different action from the smaller, producing relaxation, instead of constriction. If then large doses of opium are relaxing, and diaphoretic, why are they not applicable in many cases of Inflammation, even of the most Sthenic grade, particularly after due depletion.

With many Physicians, this may conflict with favorite views of the Pathology of Inflammation. With me such speculations are of little moment. Whether Cullin was right in believing that spasm of the extreme vessels be the proximate cause of Inflammation, on that of Dr. Philip who supposed it to be the result of "Debility" of the capillaries incapacitating them for the transmission of the blood or the theory of John Hunter, who believed it to be the result of increased "vital activity" is in the present state of our knowledged unsettled; they are questions that probably never will be definitely settled, and even if they were, would be of but little practical utility as they refer to mere local action, without any reference to the increased action of the Heart, and larger vessels, or to the altered constituents of the blood.

But as time will not further allow the consideration of Inflammation in general, we come now to its applicability in Individual Diseases. In the Treatment of Pneumonia, the attention of the Profession has been recently called to the powers of opium as a curative agent. And here I may say, that there is probably no Disease in which more opposition has been arrayed against its use than in Pneumonia, but this opposition has been based upon purely theoretical grounds, and from incorrect views of the operation of the remedy. If Physicians were to discard much of their theory, or at least make it of secondary consideration and confine themselves to a more thorough and systematic investigation of certain remedies to their cure, there would certainly be less confusion in the profession. My object then is to present the reorded testimony of eminent and experienced men who speak strongly in its favor.

By a reference to the *N. W. Med. and Surg. Journal*, I find that Prof. Herrick is one of its supporters. He uses it in large and repeated portions in pneumonia, in connexion with quinine. Hear what he says: "The almost uniform success of this treatment in our practice for several years, has been such, that we can now recommend it to our readers with confidence." He then accompanies this with the history of several cases, showing his mode of treatment, that rapidly recovered under its use. In the November number of the same Journal for 1852, the same author says of opium in pneumonia, "that given in full doses, by which we mean from 3 to 6 grains, repeated from every 4 to 8 hours, it not only modifies the respiratory and circulatory movements, but acts upon the excretory organs, as the skin, kidneys, etc." He follows this with a case of pleurisy, complicating pneumonia, in which this remedy was given, and in two days from the commencement of its exhibition, the case was convalescent; such evidence emanating from such a man as Prof. Herrick, is entitled to much weight. Prof. Austin Flint, to whom I have already alluded, I find, by reference to my book of notes, speaks highly of the use of opium combined with Dover or some other diaphoretic in pneumonia. I am inclined to the belief myself, that in most cases of inflammation of a high sthenic grade, opium is best administered in combination with ipecac or tart. of antimony, as we thus secure its diaphoretic effects without the necessity of giving it in such large quantities, believing, as Dr. Henry, that "its good effects are attributable to its powers of sedation, but mainly to its diaphoretic properties."

Dr Chas. L. Duncan, of this place, and a member of this society, informs me that he has made opium the basis of treatment in pneumonia, for several years past. He has kindly furnished me with the following case:

"Was called upon one evening to see a child of Mr. B., age 8 years. Symptoms were high fever, hurried breathing, pain in the right side, flushed face, tongue furred, considerable cough, with but little expectoration. As the bowels were bound, ordered an emeto-cathartic of cal. ipecac.

Second day visited the case and found but little improvement, although the cathartic had operated well. Gave opium gr. 1, and ipecac gr. $\frac{1}{2}$ in powders, to be given at intervals of two hours through the day. Saw the case in the evening with but little alteration; ordered the same to be continued.

Third morning there was great improvement; there was but little fever, the difficulty of breathing was relieved, no pain in the side; the medicine was gradually withdrawn, and the patient made a rapid recovery."

In this case the disease was certainly arrested before it had progressed to the second stage.

In peritonitis, according to the experience of some, opium in large doses, is especially applicable, although at variance with the well settled opinion of the most of the profession. Prof. Austin Flint is one of the advocates of the remedy. By a reference to my book of notes, taken from him whilst lecturing upon this disease, in speaking of the use of cathartics, he makes use of the emphatic language that they are "positively injurious, that they prove pernicious by increasing the peristaltic motion of the bowels, and that the indication is to keep them quiet, and as far as possible to control inflammatory action, and that this cannot be accomplished in any better way than by large and repeated portions of opium." Is not this mode of reasoning rational? For myself, although I have not fully tested its virtues in this disease, I am convinced in reason of its correctness. The same author remarks, that under this treatment, "although the disease is grave in its character, that there is no one of equal gravity so under the control of medicine."

In that fatal disease, puerperal fever or puerperal peritonitis, when of the sthenic grade, Dr. Henry Miller, Prof of Obstetrics in the University of Louisville, employs opium freely, after depletion of blood letting, but considers purgations out of the question, as worse than useless.

In the asthentic variety of the same disease, he makes use of the strong assertion, based upon the ex erience of years, that it is not called for, nor will it bear the loss of blood or depletion of any kind;" but that opium should be used liberally as affording the only chance of safety.

Dr. Clark, of New York, has pushed this remedy to a greater extent probably, than any man of his day. I cannot do any better than transcribe one of the cases, taken from a lecture of Dr. Clarks, by Edward Cook, M.D., in his Inaugural Dissertation, and published in the Western Journal of Medicine.

"First Case—first day—announced by a chill, pulse 120, skin hot, tongue clean, respiration thoracic, abdomen tympanitic. Dr. Smith, of Bellevue, gave, according to Dr. C.'s instructions, ten miniums of magendies solution of sulphate of morphia.

Second day—Pulse 120, skin hot, tongue not quite so clean, respiration thoracic, abdomen more tympanitic, vomiting of a greenish stuff like bile. One grain of sulph. morphia every hour; pulse slightly reduced.

Third day—Symptoms of second day still continuing; 1⅓ gr. of morphia every hour, slight narcotism induced, pulse fell to 90, the respiration to 7, vomiting continued, but the greenish color disappeared so soon as narcotism came on; 3 grains of opium are now ordered every hour. The effects were deeper narcotism, disappearance of the tympanitis and tenderness, fall of the respiration to 5, vomiting still persistent.

Fourth day—Diminished doses of opium, 1 gr. every two hours, continued for four days, surface moist, tongue slightly furred, no tenderness, no tympanitis. After the eighth day a dose of oil was given. The lochia were checked before the attack, at its commencement the milk secretion. For the first four days the patient averaged 4 grs. of opium every hour."

In a second case where the symptoms were still more unfavorable, it was used in still greater quantities. Dr. Cook adds "These were both cases of puerperal peritonitis; the second inordinately severe and unfavorable; both were treated by opium alone: both terminated well.

It will be seen that Dr. Clark does not give opium by quantity, but for effect, and the report of his cases show, that he has met with better success in its treatment, than any other man whose experience is on record.

Of the superior efficacy of opium in dysentery the profession are daily becoming more united; but it still has, as in the days of Sydenham, its opponents. That great and good man, acknowledged to be the most illustrious of his day, as a close and accurate observer of the operations of nature, notwithstanding the theory of that time was in opposition to its use, regarded it, as did his successor, Dr. Armstrong, who considered it of indispensable value.

Prof. Austin Flint, to whom I have made frequent mention in these pages, sustains its use in dysentery as in some other forms of inflammation. He uses it in both the sporadic and epidemic forms of the disease. His treatment is not exclusive, in the first place he unloads the bowels with castor oil, or some of the saline cathartics, and follows this with free and repeated portions of opium, and continues its use for several days if necessary, at which time if the Dysenteric discharges still continue, he again opens the bowels with an aperient, and immediately upon its action resumes its use.

It would be doing injustice to the high reputation of Professor Flint, or in fact any of the advocates for the use of opium, to say that they have adopted a rotine course, or did not adopt their treatment to the various modifications of the disease, or the circumstances of each individual case.

Would they if it gave evidence of a decided Inflammatory character, refrain from the benefit to be derived from blood-letting? Or would they in Epidemic Dysentery, where a powerful depressing agent was at work, making the disease of a decided adynamic character withhold the use of nourishing and sustaining remedies?

Or when the disease partakes of the Intermitting form, withhold the use of Quinine? And even in cases where there was strong presumption that the liver was at fault, withhold the use of Calomel? I can speak myself; certainly not. But that opium should be the leading remedy I believe, as it *relieves pain*, *quiets the bowels*, and controlls *Inflammatory action*, which I conceive to be the great indications in its treatment. The learned author referred to above in speaking of the use of cathartics in this disease, as in peritonitis, says "that they must occasionally be used, but that their direct operation is bad." Mercury he considers in many cases does harm, that its good effects as an Antiphlogistic does not compensate for the injurious effect it has upon the Inflamed mucous membrane. That it has a specific action upon the liver there are but few that will deny, and it is for this that many advocate its use in Dysentery, believing that the liver is at fault; but such I in candor believe to be in error. Such individuals object to the use of opium on the ground that it increases the Inflammation as well as stops the secretion of bile. That the first is not the case we have the testimony of many eminent men, such as Stokes, Cheyne, Watson, Williams, and of our own country, Flint, Clark, Herrick and many others. That the last is true I have seen from my own experience. But to increase this effect, it should be given in large portions. Dr. Armstrong, years ago said "that wherever opium is given in any abdominal Inflammation the dose 'should be large, for a small dose often stimulates, whereas a large one is directly sedative."

That the action of cathartics impair the power of opium I have frequently observed. Dr. Henry thinks the bowels should be kept quiet some 36 hours before giving a purgative. He says that if given too soon "you are almost certain to reproduce the disease, and involve the necessity of commencing anew with your treatment."

I have in my possession the journals containing the experience of men living in different parts of our country, who have had extensive experience in this disease, who speak in unqualified terms of the power of opium. To instance, in the Southern *Med. and Surg. Journal*, is some remarks on the treatment of Dysentery, by E. F. Starr, M. D. He says "But to come directly to the question—What is the remedy for this disease? I answer emphatically opium. This shovld be considered the leading remedy in its treatment. The chief properties of opium are to relieve pain, produce somnolency, and cure Inf. of the mucous membranes, and I may add of most of the other tissues."

In the November No. of the *North West Med. & Surg*

*Journal* for 1852, is an abstract of some remarks made by A. B. Palmer, before the Cook County Medical Society in which he says "Opium is an indispensable agent in the correct treatment of Dysentery, and should be used from the beginning to the end of the disease, much of the time in connexion with other means, he depends upon it to allay pain, procure rest, cause diaphoresis, and by a direct action which opium is capable of, to subdue Inflammation. He gives from 2 to 4 grains, sufficient to produce a decided Anodyne influence."

It may be said that I am too warm in my attachments to opium, but I would not advocate its claims so strongly were it not based upon the judgement and experience of many of our ablest men, and this again strengthened by my own experience. I have tested its virtues perhaps more thoroughly in Dysentery, than in any other disease, and am satisfied in my own mind, that it possesses greater curative powers, than any one agent we possess; and although I should not like to be restricted to any one remedy, in any given disease, still if I had to make my selection, in this disease, I should without hesitation select opium.

I have not been long in this belief, as I formerly entertained the same view in common with most physicians that the disease was secondary, and that to give opium without being combined with Calomel, to keep up the secretions, and a constant excitement of the liver, would be a grievous error, and one quite unpardonable, and it was not until I noticed the ill success of others, and experienced it myself, that I was satisfied of its inefficiency. It is true however, whilst we advocate its superior claims, we cannot claim for it the merit of curing every case, for it is well known to every intelligent Physician, that in many cases the powers of life are so overwhelmed by the shock of the disease and the tendency to fatality so great that no human cause however potent has been found sufficient to prevent a fatal issue.

Such was the nature of the Epidemic that visited this town and vicinity the last summer. Most of the cases were of a malignant Typhoid type, attacking infants and children under 2 years of age, cases that could not tolerate the remedy as those more advanced in years.

But there were cases in which it was well tested and with the most happy effects, and to illustrate the course I pursued and the nature of the disease, I cannot do better than report one of them.

Case 1. Patient 8 years of age. Was called to see this case the evening of the 2nd day after the Disease had been fully formed. I learned from the family that it was preceded by a Diarrhœa with some intermixture of blood several days previous to the Dysenteric symptoms. The patient had been given medicine but followed by no relief. Symptoms at this time were, Pulse frequent and feeble, timed by the watch 130 per minute, great tenesmus and pain in the bowels. Dejections, at times copious, at other times none, and as often as every 5 or 10 minutes—Tongue was of a dark brown appearance in the centre and red around the edges. Patient had frequent sinking spells. There was considerable gastric irritation, a deep red spot was on each cheek, a purple appearance of the eyes. The expression of the countenance was dull and languid, abdomen was tumid, and the temperature of the skin below the natural standard. I gave the opiate pill grs. $2\frac{1}{2}$, and followed in 2 hours with powders composed of Sulphate Morphine grs. 4, pulveris doverii grs. 3, Acet Lead grs. 3—and directed them to be used alternally every 2 hours, through the course of the night. I also ordered slippery elm water as a drink.

2d day—In the morning saw the case, was not so restless, rested better through the night, the dejections were not so frequent; in other respects, but little improved.

Ordered the same to be continued with the exception of acet. lead, which was withheld. Ordered hop poultice to the bowels. and more nourishing diet.

In the evening found the pulse some fuller, respiration some reduced. Discharge from the bowels still diminished in frequency and quantity. Patient had two or three feculent discharges through the day. Directed the pills and powders to be given at intervals of 3 hours.

3d morning—The dejections were more frequent with considerable pain, and an apparent aggravation of other symptoms.

Ordered the same medicine as in the first instance.

Returned in the evening, and found the patient more comfortable, pulse reduced to 120 per minute, and the respirations much reduced, had some 3 or 4 feculent discharges, more or less mixed with bile. At this time the patient was troubled with an itching of the nose and face. Lessened the quantity of opium, with instruction to resume its use if necessary.

4th Morning—Patient had rested well through the night, tongue looks better, some nausea, breathing easier, much disposition to sleep—continued the remedy as above.

Evening returned and as there had been no feculent discharge, and but little blood, ordered an Aperient of Castor Oil with Spts. Terebinth, 10 drops, as the abdomen was still swollen, and directed them to resume the opium after the operation of the medicine, every three or four hours if necessary.

5th Morning—Pulse about 100 full, Tongue cleaning, the Aperient had operated well, the pills were continued every 4 hours. In the evening the patient was troubled with severe tenesmus, which was relieved by laudanum and starch injections; from this the case gradually recovered. It will be observed that I gave no Aperient which I am in the habit of doing every 36 hours, deeming it in this case unnecessary, as with one exception, there had been feculent discharges each day. I had intended to have reported another case not so decidedly asthenic, treated with slight variations from this one, but as this essay is already longer than I intended I forbear.

