# Correction

**Published:** 1854-10

**Authors:** 


*Correction.*—Our attention has been called to a typographical error in the article of Dr Payne on Opium. &c., published in the July number of this Journal. On page 300, line twentieth from the top, for "sulphate morphine, grs. iv.," read "sulphate morphine, grs. $\frac{1}{4}$." We regret that such an error should have occurred; but we presume it has occasioned no harm, as it must have been evident to all that it was an error of the types. J.

